# Sub-Frame Evaluation of Frame Synchronization for Camera Network Using Linearly Oscillating Light Spot

**DOI:** 10.3390/s21186148

**Published:** 2021-09-13

**Authors:** Hyuno Kim, Masatoshi Ishikawa

**Affiliations:** Data Science Research Division, Information Technology Center, The University of Tokyo, Hongo 7-3-1, Bunkyo-ku, Tokyo 113-8656, Japan; ishikawa@ishikawa-vision.org

**Keywords:** frame synchronization, camera network, computer vision, linear regression

## Abstract

Precisely evaluating the frame synchronization of the camera network is often required for accurate data fusion from multiple visual information. This paper presents a novel method to estimate the synchronization accuracy by using inherent visual information of linearly oscillating light spot captured in the camera images instead of using luminescence information or depending on external measurement instrument. The suggested method is compared to the conventional evaluation method to prove the feasibility. Our experiment result implies that the estimation accuracy of the frame synchronization can be achieved in sub-millisecond order.

## 1. Introduction

Frame synchronization is often regarded as an important factor for data fusion in multi-camera systems and camera networks, and various studies have been conducted [1,2,3,4,5,6,7,8,9,10,11,12,13]. In many practical cases [14,15,16,17,18,19,20], such as tracking and monitoring on sports [14,15,16], human motion [17,18], surveillance systems [19], etc., more precise frame synchronization promises more accurate data processing results. Therefore, the evaluation of the accuracy of the frame synchronization should be carefully advanced before the endeavor to improve the quality of the synchronization itself. The way to evaluate the frame synchronization can be divided into two categories: the direct measurement of the relevant physical signals issued from cameras and the estimation using inherent information in the captured images.

The first category, the direct measurement, is the straight-forward method where each camera in the camera network issues a signal at the same moment when image acquisition starts, and the external instrument, such as an oscilloscope or multi-channel analyzer, retrieves the signals from all cameras as shown [21]. By comparing all the retrieved times, the accuracy of the synchronization is directly measured. While this kind of method can provide a high resolution of the measurement result, it imposes cumbersome wiring labor to carry the signals from cameras to the instrument and often requires an expensive multi-channel analyzer. Therefore, the larger scale of camera system requires more effort for the measurement, which is regarded as a disadvantage when applying this method.

On the other hand, the second category, the estimation using the captured images exploits inherent information of a visualized target in the captured images [1,2,3,4,5,6,7,8,9,10]. A light source is often used to produce visual information to evaluate the frame synchronization [1,2,3,4]. For example, a blinking light source is the simplest way to generate the information, as reported in our previous work [1]. When the frequency of blinking is higher than the frame rate of a camera, it is possible to evaluate the frame synchronization at the measurement resolution which is equal to the time interval of two adjacent images. If more precise estimation, such as subframe level, is required, a more specific way, such as that suggested by L. Hou et al. [2], can be adopted, where the illumination of the light source is temporally modulated, and then the resolution of estimation increases up to tens of microseconds. However, modulated illumination is apt to be affected by surrounding light conditions and requires special illumination equipment that produces specially designated luminescence patterns.

**Figure 1:**
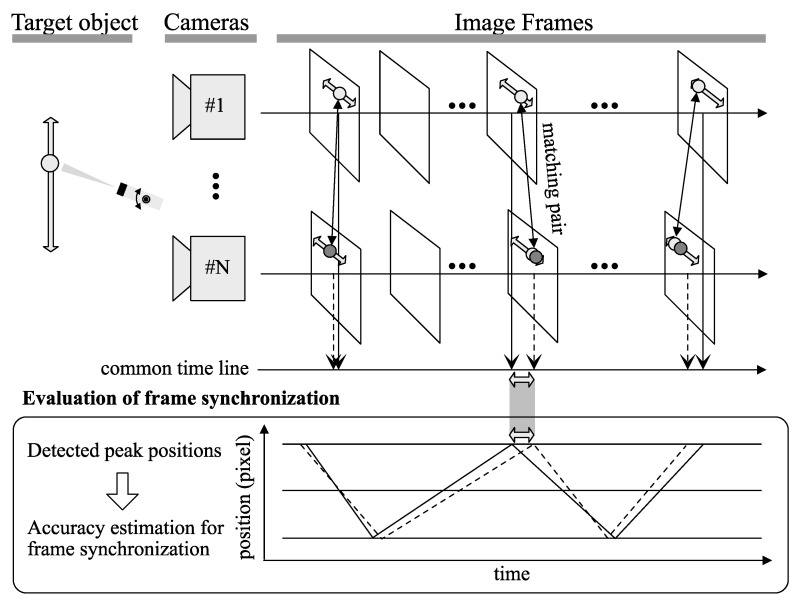
Suggested concept to evaluate the frame synchronization. Temporal skew of corresponding images captured by multiple cameras is shown as the position shift of the target object in each image frame. By detecting the position shift, the temporal skew that indicates frame synchronization can be estimated. This paper deals with how accurately the frame synchronization can be estimated by producing legible shift patterns and detecting the extent of the shift.

This paper suggests a simple and precise alternative method to evaluate the frame synchronization of multiple cameras based on inherent information regarding the position of a light source instead of its luminescence, where the position of the light source in the image plane linearly oscillates. Because the suggested method does not require regulation or modulation of the brightness of the light source, it is expected to be more robust against the surrounding light condition. Figure 1 shows the concept of our proposed method.

## 2. Frame Synchronization

### 2.1. Evaluation of Frame Synchronization

Each camera consisting of a multi-camera system or camera network is supposed to capture an image frame according to its designated time schedule. Generally, all the image frames acquired from all cameras at a certain moment are arranged at the same timeline and synchronized to be processed simultaneously, which ensures the homogeneity of visual information gathered through multiple cameras.

The extent of synchronization is evaluated as the time variance of all image frames located in a common timeline. Considering the same exposure time of all cameras, the start time or end time of camera shuttering can be exploited as the representative time that indicates temporal synchronization for the image capturing. Because most of the off-the-shelf cameras provide the function to issue a signal as an electrical pulse according to the exposure event, monitoring the pulse is the easiest way to evaluate frame synchronization. Although this is a straightforward and very accurate way, it is cumbersome to build up a wiring path for the measuring, especially when the scale of the camera system enlarges. For that reason, in many practical cases, the software-based measurement or the image-based estimation methods using the inherent information is preferred. The details will be discussed in the next subsection.

The frame synchronization of multiple cameras is dealt with statistically, where the time variance of the image acquisition that is described by standard deviation and the difference between its maximum and minimum time are often utilized to evaluate the accuracy of the synchronization quantitatively. Although the accuracy also varies over time, it is expected to spread in a statistical distribution and be indicated by a representative value. Because the exposure time of a camera is controlled by the hardware and considered approximately constant, any edge time of the exposure event can be used for the synchronization evaluation.

### 2.2. Related Works

The evaluation methods that use the scenery information in the captured image except for the direct measurement using the physical signal have been preferred for the frame synchronization due to the convenience for the practical applications. The simplest way to evaluate the frame synchronization is to check whether all the camera images captured at the same moment include the same scenery. The blink of the light is frequently exploited in this way [1], where the on-off state of the light is controlled so that the on-off period is shorter than the time interval of the adjacent two images. It is easy to adapt while having the drawback that the resolution of estimation can be less than the time interval between the two images. For precise estimation, more sophisticated methods, such as in Refs. [2,3,5], are required, where temporally encoded illumination [2] and accurate control of exposure time [3] help achieve the sub-frame accuracy of synchronization. Besides the aforementioned methods, there also exist various types of methods for frame synchronization. For example, spatial trajectory matching [6], silhouettes in videos [7], temporal signals from epipolar lines [10], camera flashes [11], and network synchronization [12] can be exploited to synchronize sequential image frames from multiple video sources as evaluating the synchronization concurrently.

Especially, among the preceding studies, the techniques using spatiotemporal information in a visual scene, as presented in Refs. [6,7,8,9,10], are often exploited due to the convenience in practical applications. In those cases, the moving objects as a target observed in camera images provide the clue to estimate the temporal arrangement of image frames directly from their motion. However, most of them require the extraction of complex image features and the matching of the corresponding spatial information over multiple camera images. Even though those methods show excellent results about video synchronization from multiple cameras, still, more quantitative and precise evaluation method with simple and fast algorithm is required depending on the practical application. For instance, a high-speed camera network [1] for a real-time visual feedback system demands sub-millisecond order of synchronization accuracy, and high-resolution 3D shape measurement [22] often requires high precision frame synchronization more accurately than the sub-frame.

Thus, we focus here on the alternative method to evaluate the frame synchronization quantitatively, which achieves sub-frame and sub-millisecond order of estimation and also has more scalability for a large-scale camera network. The suggested method is based on linear regression, which is regarded as an effective tool for analyzing sensor network data [23]. We want to emphasize here that this work is not to show performance comparison to various alternative methods. Many of those methods require dedicated equipment not easy to prepare or do not profoundly focus on the quantitative evaluation to the ground truth. The goal of this work is to prove the feasibility of the alternatively suggested method, comparing the performance with ground truth.

## 3. Methodology

Linear motion of a light spot in the physical world is also projected to linear motion in the image plane of a camera. When the light spot repeats reciprocating motion on the linear path between two certain points, the points are locally observed as the peak points of position over time in a time series data. Considering that the whole cameras of a camera network are completely synchronized and capture the same scenery into images at the same time, the timestamps of these peak points will appear on the identical position in the timeline for all cameras. On the contrary, somewhat skewed synchronization produces the temporal difference of the timestamps. Therefore, for practical cases, investigating the difference leads to the evaluation of synchronization. If it is possible to specify the peak points of the time-series data, the time distribution represents the frame synchronization on the whole cameras. Here, we suggest a method on how to produce appropriate peak points in the image plane and how to precisely estimate its position in the timeline to evaluate the frame synchronization, as shown in Figure 1.

The temporal position of peak points can be detected by differentiating the time series data after some noise filtering, where the differential value equals zero. Therefore, the shape of the curve around the peak point is very crucial to determine the accurate position of the peak point, and a more acute angle at the peak point consisting of a local triangle helps to specify a more accurate position of the peak. However, in many practical cases, it is not easy to get the angle acute enough to specify the actual peak due to the various types of measurement noise. To adapt a triangle wave as the trajectory of the light spot in the image plane could be a solution to satisfy the preceding condition. The triangle wave in ideal condition produces an acute angle around a peak point as a vertex. Even if the condition is corrupted by noise, then, we can easily find the vertex by an alternative way using linear regression for the data between two adjacent peak points. Because a triangle wave always includes a line between two vertices, it is expected that our measurement data are distributed near the line even under the existence of some noise. Therefore, after approximating the linear sections of the triangle wave to lines with linear regression, the peak position is determined as the intersection of two adjacent lines. Since it is mathematically calculated, the position features the accuracy of sub-frame resolution.

Therefore, the suggested method consists of the following four major processes: (1) projection of triangle wave using a light spot, (2) acquisition of the centroid of the light spot in the image plane, and generation of its trajectory to produce time-series data, (3) extraction of the linear section of the data, and (4) calculation of the line properties of the linear sections with the linear regression, as well as estimation of the intersection of them. The details of these processes are discussed in Section 3.1, Section 3.2, Section 3.3 and Section 3.4.

### 3.1. Projection

A light spot is projected to a screen, reciprocating between any two points on a line at a constant speed, so that its trajectory draws a triangle wave over time. Assume the line is parallel to a basis of the image plane to make the mathematical process simple. Selecting *x*-axis as the basis, the triangle wave can be defined as follows:

(1) $$x\left(t\right)=\frac{2A}{\pi }\arcsin\left(\sin\left(\frac{2\pi }{T}\left(t-\delta \right)\right)\right),$$

where *A* is the amplitude of the triangle wave, *T* indicates the period, δ means the phase shift, and 2A equals the distance between the two points.

**Figure 2:**
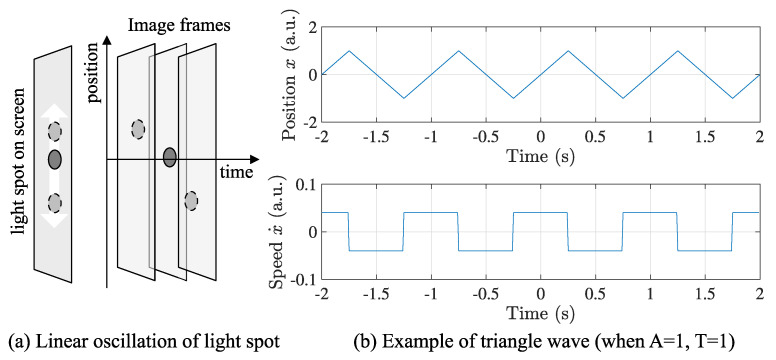
Linear oscillation of light spot and triangle wave. (**a**) The position of light spot linearly oscillating on a screen produces (**b**) a triangle wave in spatiotemporal space. In the triangle wave, the temporal positions of peak points play the key role to evaluate the frame synchronization quantitatively, which is equivalent to the edges of the square wave as the differential of the triangle wave.

Although the amplitude *A* may vary each cycle, it is regarded as a constant here to make the post-data processing simple. During a half cycle, the speed of reciprocating motion, $\dot{x}$, is kept constant. Figure 2 shows the triangle wave generated by Equation (1) when A=1, T=1, and δ=0.

### 3.2. Data Acquisition and Trajectory Generation

For generating the trajectory of a light spot, the centroid of the light spot should be determined in the image plane in advance. Since the light spot is considered far brighter than its surrounding, the centroid is easily acquired by calculating the image moments after the image binarization with an appropriate threshold value [24]. Let the threshold $I_{thr}$, and then the binary image is acquired as follows:

(2) $$I_{b}\left(x,y\right)=\left\{\begin{array}{cc} 1 & \mathrm{if}\;I_{b}\left(x,y\right)\geq I_{thr}\\ 0 & \mathrm{if}\;I_{b}\left(x,y\right)<I_{thr} \end{array}\right.,$$

where I(x,y) is the pixel intensity at position C(x,y) in the image coordinate. With Equation (2), the image moments are calculated as in:

(3) $$\begin{array}{c} M_{00}=\sum_{x}\sum_{y}I_{b}\left(x,y\right)\\ M_{10}=\sum_{x}\sum_{y}xI_{b}\left(x,y\right)\; \end{array}.$$

Then, the position *x* in Equation (1) is calculated by using Equation (3):

(4) $$x=C_{x}=\frac{M_{10}}{M_{00}}.$$

**Figure 3:**
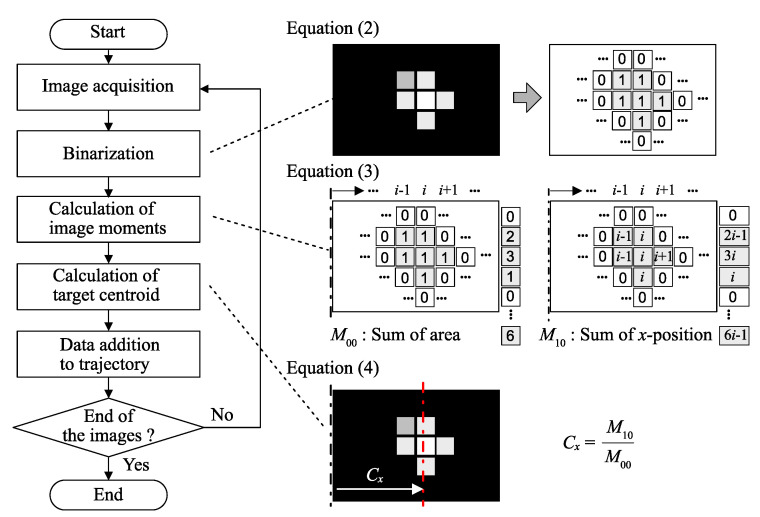
Flowchart for trajectory generation. The centroid of the light spot is calculated by simple image processing, such as the binarization and image moments calculation at every image frame. Then, the trajectory of the centroids is retrieved as the time-series data.

Therefore, the trajectory $x=\{x_{1},\ldots ,x_{i},\ldots ,x_{n}\}$, where the image index *i* increases from 1 to *n*, is calculated using Equation (4) as a function of discrete time $t=\{t_{1},\ldots ,t_{n}\}$ for *n* time-series images. When the light spot is projected on the screen according to Equation (1), then we can reconstruct the corresponding trajectory x from Equations (3) and (4). Figure 3 shows the whole process to generate the trajectory.

### 3.3. Feature Extraction

**Figure 4:**
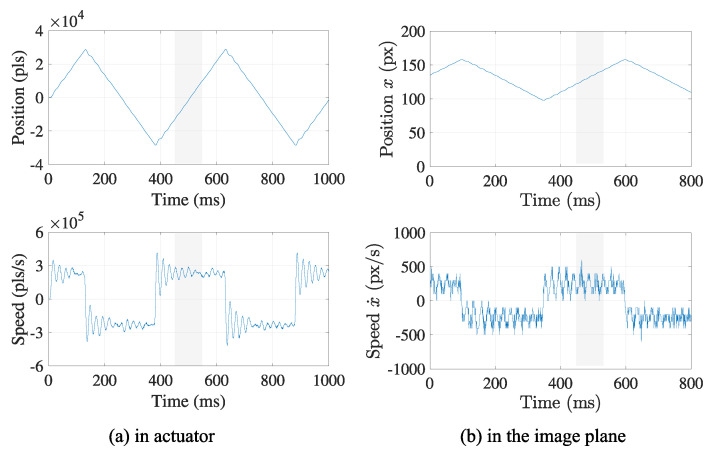
Example of a triangle wave achievable in the practical case. The retrieved trajectory is apt to be deteriorated by the practical limitation of the actuator control. The outward peak positions generated by an actual actuator are shifted by the noisy measurement result due to the inertia of the system and friction force, etc., which hinders detecting the true positions of the peaks.

In a practical case, it is difficult to control x(t) as exactly the same as defined in Equation (1), especially around its vertices, as shown in Figure 4. The inertia of the physical equipment, such as a motor, and the rapid inversion of the moving direction hinder from following the planned trajectory. Thus, trajectory x around any vertices can be disrupted by the control error, as well as the measurement noise, in the calculation of Equation (3). Although noise filtering can alleviate these aspects, determining position of the vertices based on local information is not always accurate due to the control error when executing Equation (1). Alternatively, the estimation using the linear section (gray region in Figure 4) of the triangle wave is expected to provide a more accurate result due to its following properties: the linear section includes more sampling data. At the section, the motor rotates at a constant speed without any direction change, which increases the effect of the noise filtering and the stability of the trajectory control.

**Figure 5:**
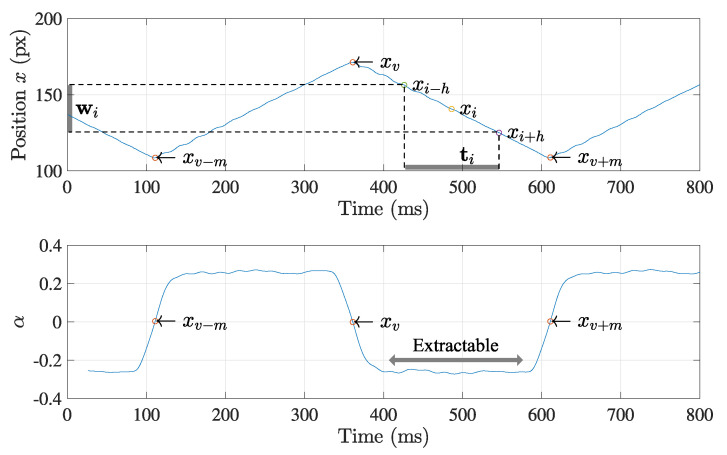
Extraction of a linear section from the trajectory. The linear section of the trajectory is extracted with the suggested method based on the linear regression. The slope α of the regression line can be a criterion to extract the linear section from the noisy trajectory.

The way to extract the linear section affects the accuracy of the position determination for the vertices. We suggest an elimination method where the only noisy data surrounding the vertices are removed from trajectory x. Let us assume that $\bar{x}=\left\{\ldots ,x_{v-m},\ldots ,x_{v},\ldots ,x_{v+m},\ldots \right\}$ is a partial trajectory, where *v* and *m* are two position indices which define three vertices $x_{v-m}$, $x_{v}$, and $x_{v+m}$, and their two linear sections. Provided that the data points around the vertices are far apart from Equation (1) due to the large control error, then, the first step is to specify the surrounding of the vertices and eliminate them from $\bar{x}$. Let a window $w_{i}=\left\{x_{i-h},\ldots ,x_{i},\ldots ,x_{i+h}\right\}$ and the corresponding time set $t_{i}=\left\{t_{i-h},\ldots ,t_{i},\ldots ,t_{i+h}\right\}$ include a point $p_{i}\left(t_{i},x_{i}\right)$ where *h* is the window size. Then, we can set the line model using linear regression:

(5) $$w_{i}=\alpha_{i}t_{i}+\beta +\epsilon ,$$

where ϵ is the error term. The residual sum of squares (RSS) regarding ϵ in Equation (5) is minimized by least squares, and we can calculate the local slope $\alpha_{i}$ at $p_{i}$:

(6) $$\alpha_{i}=\frac{\sum_{j=i-h}^{j=i+h}\left(t_{j}-E\left(t_{i}\right)\right)\left(x_{j}-E\left(w_{i}\right)\right)}{\sum_{j=i-h}^{j=i+h}{\left(t_{j}-E\left(t_{i}\right)\right)}^{2}},$$

where E() indicates the sample means of the parameters in the parenthesis. We could extract the linear section by using the local slope $\alpha_{i}$ as an extraction criterion, as shown in Figure 5.

Note that, theoretically, the local slope $\alpha_{i}$ in Equation (6) of each linear section should be constant due to the property of the triangle wave. Provided that the constant equals $\alpha_{c}$ for the partial data set $\left\{\alpha_{v-m},\ldots ,\alpha_{v}\right\}$, then $-\alpha_{c}$ is also constant for the rest data set $\left\{\alpha_{v},\ldots ,\alpha_{v+m}\right\}$. Because the slope $\alpha_{v}$ at the vertex $p_{v}$ is expected to be approximately zero, it is an easy task to specify $p_{v}$ and eliminate its surroundings to produce the linear sections of the trajectory $\bar{x}$.

### 3.4. Estimation of Frame Synchronization

Now, two adjacent linear sections are prepared, from which the equation of two lines are estimated by least squares method, as in the preceding. Let two data sets of the linear parts be $l_{k}=\left\{x_{c-w}^{k},\ldots ,x_{c}^{k},\ldots ,x_{c+w}^{k}\right\}\left(k=1,2\right)$, where *k*, *c*, *w* are the indices specifying the linear section, indicating the center of the linear section, and representing the half windows size of the linear section, respectively. Then, the regression model of them can be described in the same manner:

(7) $$l_{k}=\alpha_{k}t_{k}+\beta_{k}+\epsilon ,$$

(8) $$\begin{array}{c} \alpha_{k}=\frac{\sum_{j=c-w}^{j=c+w}(t_{j}-E\left(t\right))\left(t_{j}-E\left(l_{k}\right)\right)}{\sum_{j=c-w}^{j=c+w}{\left(t_{j}-E\left(t\right)\right)}^{2}}\\ \beta_{k}=E\left(l_{k}\right)-\alpha_{k}E\left(t\right)) \end{array}.$$

Therefore, the *t*-coordinate of the intersection of two linear parts $l_{1}$ and $l_{2}$, $t_{int}$ is calculated using Equations (7) and (8), as follows:

(9) $$t_{int}=\frac{\beta_{1}-\beta_{2}}{\alpha_{2}-\alpha_{1}},\;\mathrm{when}\;\alpha_{2}\neq \alpha_{1}.$$

**Figure 6:**
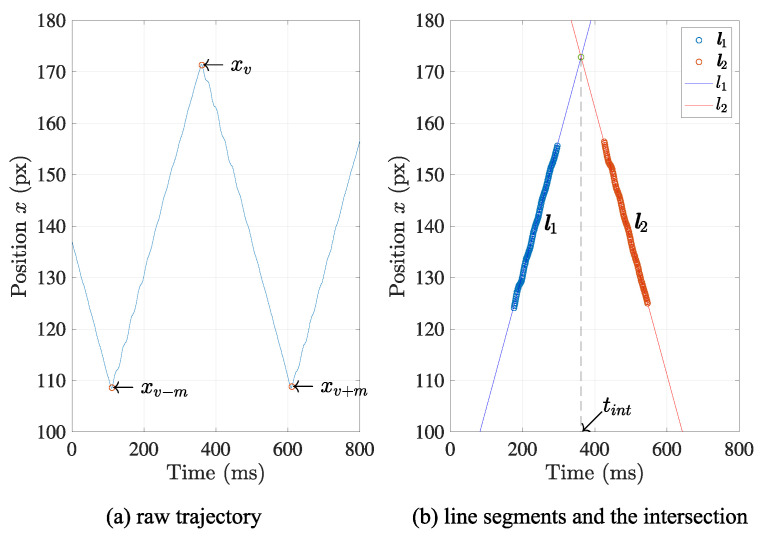
Estimation of the temporal feature point. The intersection of two extracted linear sections is regarded as the temporal feature point to represent the frame synchronization.

The $t_{int}$ in Equation (9) can be regarded as a temporal feature point that indicates a specific moment when the moving direction of the light spot changes. Figure 6 represents these processes with visualization.

Through the processes in Section 3.1, Section 3.2, Section 3.3 and Section 3.4, the frame synchronization of the multiple cameras can be evaluated with $t_{int}$ from the visual information in the captured images. Let the camera index be *j* and the intersection for each camera $t_{int,j}$, and then $t_{int}=\left\{t_{int,1},\ldots ,t_{int,j},\ldots ,t_{int,n}\right\}$(j=1,…,n) is regarded as the data set of different timestamps for the same event taken by multiple (*n*) cameras. Consequently, the accuracy of frame synchronization is estimated with the MAX-MIN value and the standard deviation of $t_{int}$. In the later part of this paper, the experiment for evaluation of the frame synchronization is conducted using the suggested method, and the result will be compared to the conventional evaluation method that exploits the physical signals issued from the cameras.

## 4. Experiment

**Figure 7:**
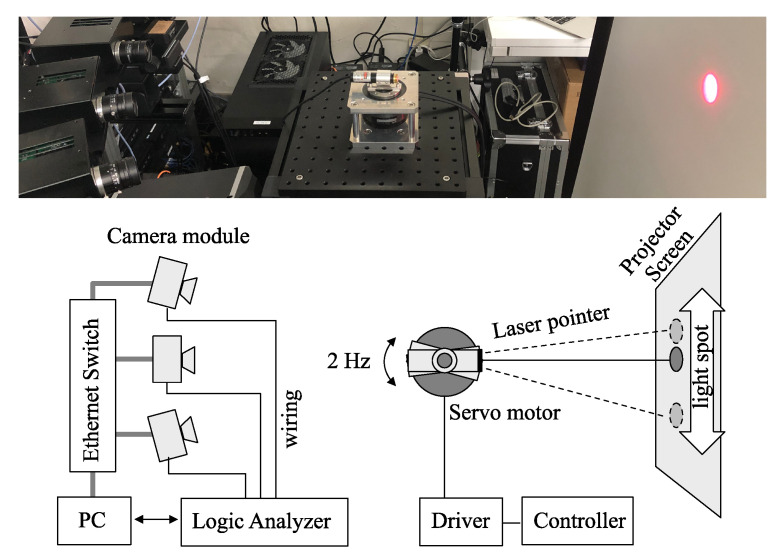
Outline of the experiment to evaluate the frame synchronization using light spot.

The evaluation method suggested in the preceding chapter was verified with an experiment where three cameras on the same camera network capture the oscillation of the light spot at the same time. The experiment system consists of a laser projector with an actuator system, a projector screen, networked cameras, and a multi-channel logic analyzer, as shown in Figure 7.

The laser projector was mounted on a high-speed motor and rotated back and forth to produce a small amount of angular oscillation that is observed as the linear oscillation on the project screen. The motor driver was operated by a controller, where proportional-derivative (PD) control was applied to produce a triangle wave of 2 Hz. The control frequency was set to 1000 Hz. As addressed in the previous chapter, the acquired trajectory of the motor can be deteriorated by the control error, external force, friction, etc. Although more accurate control of the motor can improve the accuracy of the evaluation, it is difficult to realize the theoretical trajectory in practical systems. However, the aspect of these inaccuracies will be absorbed by the suggested method in the evaluation processes.

Three cameras on a camera network were synchronized by the methodology using the reference broadcast scheme presented in our previous work [1,25] to capture the light spot on the screen. The accuracy of synchronization is expected to be less than 1 millisecond and the frame rate of the camera was set to 1000 frames per second (fps) to match between them. The image capturing was conducted for 1 s, where 1000 image frames of 320 × 240 resolution are generated. After capturing a time series of images, those images were saved on the local storage of the camera and gathered by a computer later for further analysis with post image processing. The acquired images were transformed into binary images with a threshold, and the image moments for the target light spot were calculated. Then, the *x*-coordinates of the light spot in the images were acquired.

**Figure 8:**
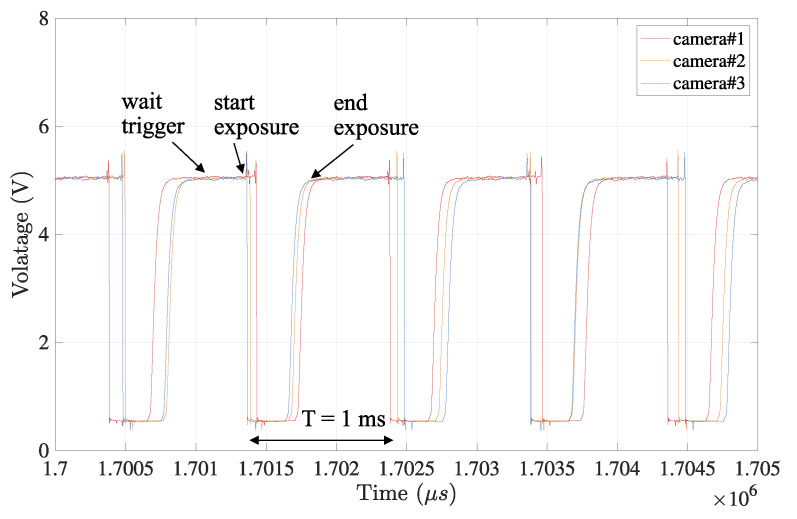
Frame synchronization captured by logic analyzer. The event times indicating exposure start and end in all cameras are monitored, where logically low state (<2 V indicates that image capturing is in progress. These times are regarded as the ground truth for the evaluation of the actual frame synchronization.

The data set of those *x*-coordinates $x=\{x_{1},x_{2},x_{3}\}$ from three cameras are separated into several linear sections for each camera, not to include the surrounding of the vertices according to the extraction process in Section 3.3. Then, from the linear sections, the temporal feature $t_{est}$ as the timestamps of their intersections, which equals $t_{int}$ as the data set of $t_{int}$ in Equation (9), were calculated using Equations (7) and (8), in the estimation process in Section 3.4. The estimated timestamps were compared to the ground truth that is acquired by external measurement equipment as the conventional measurement method. The corresponding timestamps $t_{mea}$ as the ground truth are measured by a logic analyzer, where the physical pulses issued from three cameras were registered on the same timeline when the exposure event starts, as shown in Figure 8.

Since only a set of $t_{est}$ was acquired per trial in our experiment setup, these processes were repeated ten times to provide the statistical analysis.

## 5. Result and Discussion

**Figure 9:**
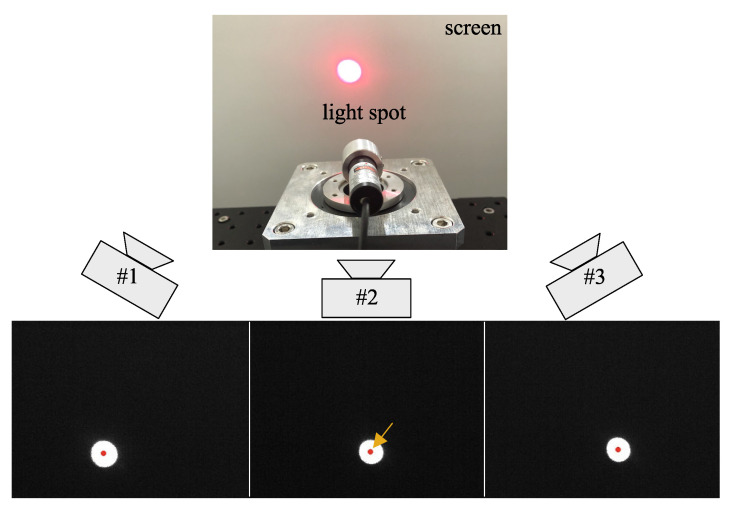
Example of image capturing and centroid acquisition. The centroid of the light spot (red dot) was clearly detected at each camera image.

An example of the captured images and the calculation result of the centroid of the light spot is shown in Figure 9. The light spot was projected on the white screen and captured by three synchronized cameras, where the centroids were clearly detected. Then, three trajectories of the centroids were retrieved for further analysis.

**Figure 10:**
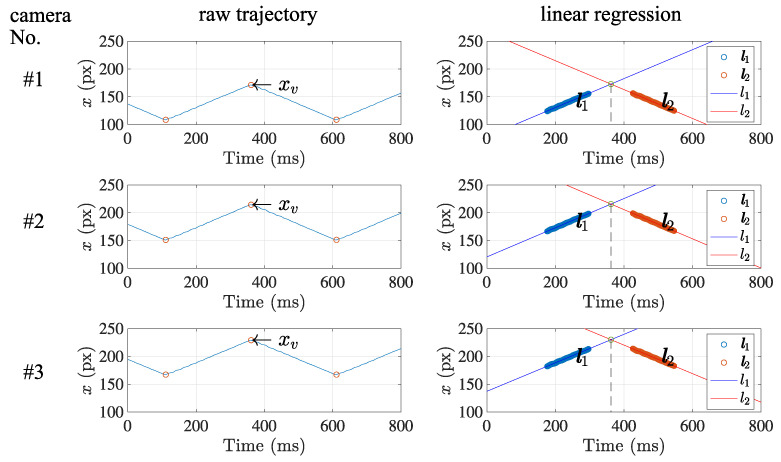
Accuracy of the estimated frame synchronization. The intersection of two adjacent linear section was calculated in sub-frame accuracy at each camera, which indicates the moment that the direction change of the light spot occurs.

Figure 10 shows the retrieved data set x and the separated linear sections $l_{1}$ and $l_{2}$. Note that the data set acquired from the centroid positions draws a corresponding triangle wave that well matches the original triangle wave generated by the actuator.

After the separation result was derived by the suggested method, the estimation of frame synchronization was conducted after finding the regression lines, as also shown in Figure 10. Then, each temporal feature point of the timestamp that represents the frame synchronization was calculated as the intersection of the two regression lines. It provides sub-frame resolution, which equals sub-milliseconds less than the time interval of frames.

**Figure 11:**
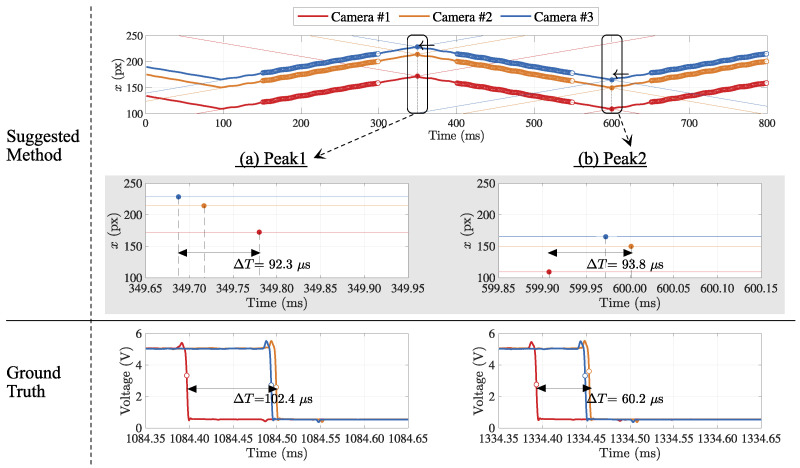
Comparison of the frame synchronization estimations by suggested method to ground truth. Two feature points were detected as the intersections of the regression lines for each camera, and three temporal feature points per each peak position were aligned. The max-min value of them was compared to the ground truth by a logic analyzer.

For the comparison with the conventional measurement, the physical signals issued from all cameras at the same moment when the exposure starts were also gathered, and the different arrival times were registered as the timestamps, as shown in Figure 11.

At each camera, two peak points as the temporal feature were detected as the intersections of the regression lines by the suggested method, and three temporal feature points were aligned on the same timeline at each peak. The difference of the maximum and minimum value of the feature points was registered as the indicator for frame synchronization accuracy. The estimation result was compared to the corresponding one acquired by logic analyzer as ground truth. Table 1 shows the two types of temporal features to compare the evaluated frame synchronization.

**Figure 12:**
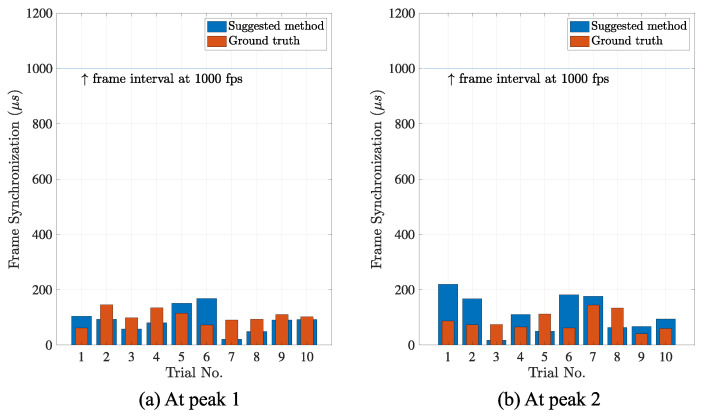
Estimated accuracy of frame synchronization. The frame synchronization was estimated by the suggested method (blue) at the sub-frame resolution. Compared to the ground truth (red), the average differences were 1.6% and 2.9% of the full frame interval 1000 μs (blue line) at two observation points (**a**,**b**). The estimation accuracy was the order of tens of microseconds.

The features are marked as the time difference between the maximum and minimum with the standard deviation in the parenthesis. The compared groups have equal variances (by F-test, *p*-values > 0.05). In both estimations, the accuracies of frame synchronization for our camera networks system were less than 1 millisecond, in the order of hundreds of microseconds. In addition, the differences of the accuracies between that by the suggested method and that by the conventional method were approximately 1.6% of the full frame interval at peak 1 and 2.9% at peak 2, in average, respectively, as shown in Figure 12. The results are considered to be enough to evaluate the sub-frame accuracy, even for the high-speed camera network that operates at 1000 fps.

The suggested method is expected to be robust against the surrounding light condition because this position-based analysis does not require any luminescent model to retrieve the temporal information for synchronized image capturing. In addition, since it exploits the inherent visual information in the camera image instead of any physical signals issued from the camera hardware, it is expected to be more suitable to adapt to more huge systems, such as wide-area camera networks, with more feasibility. On the other hand, because how to set the window size *h* of $w_{i}$ in Equation (5) and *w* of $l_{i}$ in Equation (7) can affect the estimation accuracy, it should be more carefully studied. For a similar reason, the effect of the image resolution on Equation (4) is also required to be quantified. Thus, because the parameter optimization regarding the extraction of linear sections under the noisy measurement and the refined numerical analysis are required further, we plan to deal with them in the future works.

## 6. Conclusions

This paper presented a novel evaluation method of frame synchronization for the camera network by using the inherent visual information in the captured scene. The contributions of this paper can be summarized as the following: First, the linear oscillation of a light spot was suggested to generate the inherent visual information from which the temporal feature of the frame synchronization was retrieved. Second, an analytic method based on the linear regression to compensate for the deteriorated measurement result was suggested. Finally, the suggested method was verified with the comparison to the conventional measurement method. The proposed method was able to accurately estimate the frame synchronization at the time resolution in the order of tens of microseconds.

The suggested method not only provides sub-frame estimation but also increases the feasibility for the large-scale camera network because it does not demand cumbersome wiring and stable lighting condition of surrounding. Although it was realized only with the triangle wave that still requires control of the wave shape, the provided method is expected to be adopted to various wave forms, which include oscillating shapes not limited to the triangle wave, with further improvement in the estimation accuracy by robust statistical techniques in future work.

## Figures and Tables

**Table 1:** The frame synchronization estimated at two peak points.

Trial No.	Max-Min (Stdev) of Timestamps [μs]			
	Peak 1		Peak 2	
	Suggested	Ground Truth	Suggested	Ground Truth
1	103.8 (58)	61.4 (33)	220.0 (125 **)	86.7 (43)
2	93.1 (54)	145.4 (73)	167.6 (84)	73.4 (38)
3	58.2 (29)	98.4 (53)	17.9 (9)	74.6 (40)
4	80.9 (41)	134.6 (76)	110.8 (57)	65.1 (35)
5	151.4 (77)	115.2 (61)	50.4 (28)	112.2 (56)
6	168.3 (85)	72.3 (41)	181.9 (91)	61.9 (33)
7	20.8 (11)	90.9 (52)	176.2 (90)	144.6 (77)
8	49.6 (26)	93.4 (54)	63.2 (32)	133.8 (67)
9	90.5 (51)	110.1 (56)	66.7 (34)	41.0 (22)
10 *	92.3 (47)	102.4 (58)	93.8 (48)	60.2 (34)

* This data is the same as the one of Figure 11. ** This large value can be dealt with as an outlier.
